# A Targeted Parathyroidectomy Using Guide Wire Technique in a Pregnant Patient with Primary Hyperparathyroidism

**DOI:** 10.1155/2009/361427

**Published:** 2009-12-23

**Authors:** E. Kandil, S. Faruqui, J. Alharash, H. Alabbas, B. Rodgers, B. Blank, B. M. Jaffe

**Affiliations:** ^1^Department of Surgery, Division of Endocrine and Oncologic Surgery, Tulane University, School of Medicine, New Orleans, LA 70112, USA; ^2^Department of Radiology, Tulane University, School of Medicine, New Orleans, LA 70112, USA

## Abstract

Primary hyperparathyroidism may cause fetal demise in pregnant patients if prompt diagnosis and treatment is not initiated. The paper describes a novel guide wire technique for a targeted parathyroidectomy, which may reduce the risk to mother and fetus and be useful in other related circumstances.

## 1. Introduction

Primary hyperparathyroidism is rare in pregnancy, but the disease causes fetal demise if prompt diagnosis and effective management are not pursued. Previous studies have demonstrated that eighty-one percent of cases of primary hyperparathyroidism during pregnancy are caused by an adenoma, twelve percent by parathyroid carcinoma, and the remainder by hyperplasia [1, 2]. Maternal hypocalcemia in the mother is caused by a physiologic state of gestational hypoalbuminemia as well as a gradual increase in demand for calcium by the fetus [3]. 

In mothers with primary hyperparathyroidism, the increase in calcium levels suppresses fetal parathyroid development, ultimately leading to hypocalcemia in the neonate. Primary hyperparathyroidism has also been shown to be associated with spontaneous abortion, stillbirth, neonatal tetany, and intrauterine growth retardation.

Primary hyperparathyroidism is a difficult diagnosis to make during pregnancy because of its low clinical incidence, clinical variability, and the difficult in applying specific instrumental exams. The gold standard for preoperative localization during pregnancy is ultrasonography of the neck, which has a sixty-nine percent sensitivity and ninety-four percent specificity. Computerized tomography, parathyroid scintigraphy, and magnetic resonance imaging are also useful, but are not typically used due to potential risks to the fetus [4]. 

It is essential for surgeons to minimize the operation and anesthesia used in the treatment of primary hyperparathyroidism. This paper describes a novel guide wire technique, which provides targeted parathyroidectomy, thereby potentially reducing the risk to mother and fetus and selected others at high risk.

## 2. Case Report

A 30-year-old African-American female was referred during her second trimester of pregnancy for surgical management of persistent primary hyperparathyroidism. The patient had a history of multiple episodes of acute pancreatitis requiring more than twenty hospitalizations for the treatment of that condition. She also had a history of nephrolithiasis, but no personal or family history of MEN syndromes and was normotensive. Prior to her pregnancy, the patient had initially underwent a neck exploration for what was diagnosed as primary hyperparathyroidism at another institution, which was unsuccessful in finding the abnormal parathyroid gland. The operative report from the first surgery was reviewed. The surgeon who performed the first operation extensively described a thorough dissection during the first operation. The dissection included bilateral neck exploration, opening both carotid sheaths, and intraoperative selective venous sampling. The patient continued to have evidence of hypercalcemia postoperatively due to persistent primary hyperparathyroidism, with a serum level of calcium of 11 mg/dL, albumin of 3.6 g/dL, and an intact parathyroid hormone level of 140 pg/mL. The rest of her blood laboratory workup was within normal limits. The preoperative localization workup was performed at Tulane University Hospital and included a repeat neck ultrasound that revealed a 1.0 cm previously missed hypoechoic mass in the tracheo-esophageal groove. CT scanning was not utilized because of the risk to the fetus.

Due to the previous failed detailed dissection, the needle localization technique was chosen for this pregnant patient. This technique offered a targeted focused approach without the need for bilateral neck exploration. After obtaining informed consent from the patient, the skin was prepped in the standard fashion and local anesthesia administered. A Homer needle was introduced under ultrasound guidance and the tip guided to an appropriate position within the mass. A 22-gauge Chiba needle was passed in a tandem fashion and aspiration of the lesion was attempted. The specimen obtained was sent for intact PTH assay. Subsequently, 0.5 mL of methylene blue was instilled through the Homer needle. Under ultrasound guidance, a hook wire was passed through the Homer needle, confirming placement of the wire tip within the lesion. Both the Homer needle and wire were left in place and secured, and the wire was taped in place. The patient was then taken directly to the operating room.

**Figure 1:**
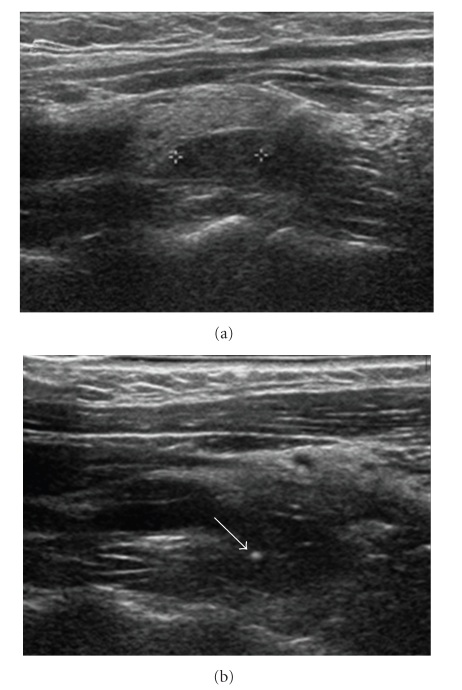
(a) Ultrasound of parathyroid adenoma. (b) Same patient, with guide wire in place (white dot).

The neck was prepped and draped in the standard fashion, including the localization wire. Surgical exploration was done under general anesthesia. A small skin incision was made which included the point of entry of the guide wire (Figure 1), and the wire was followed with meticulous dissection and guidance by the presence of methylene blue until the parathyroid adenoma was identified. All the tissue impregnated with methylene blue was excised, along with the mass containing the hook wire. PTH serum levels, monitored intraoperatively, decreased from 143 to 23.6 pg/mL after ten minutes following the removal of the mass. This significant drop confirmed the surgical correction of her disease and eliminated the possibility of hyperplasia. The procedure was done in seventy-five minutes including the waiting time for reporting the results of the intraoperative PTH assays monitoring. The patient tolerated the procedure well. Throughout the procedure, the fetus showed good movements by ultrasound. The patient did well and was discharged home the next day. The patient′s postoperative course was uneventful and she has maintained normocalcemia for six months postoperatively. 

## 3. Discussion

Primary hyperparathyroidism is a relatively common disease with an incidence of 0.1% to 0.2% in the general population. This disease occurs in all ages, but most commonly in the sixth decade of life with a three-to-one female-to-male ratio. Benign adenomas are found in approximately eighty percent of patients, and fifteen to twenty percent of cases are due to hyperplasia. The most uncommon presentation of primary hyperparathyroidism is parathyroid cancer, which is found in only 0.5% of cases [1].

Complications, including hyperemesis and nephrolithiqsis, occur in sixty-seven percent of pregnant females diagnosed with primary hyperparathyroidism [3, 5, 6]. 

More severe maternal complications, such as pancreatitis and hypercalcemic crisis, may also occur in the mother, resulting in fetal growth retardation [5, 7–9]. Other complications include spontaneous abortion, maternal death, induced labor, intrauterine growth restriction, low birth weight, and neonatal hypocalcemia [7, 10, 11]. 

The most serious complication in primary hyperparathyroidism in pregnant women is hypercalcemic crisis, defined as a serum calcium level of >14 mg/dL. This entity presents with severe nausea, vomiting, dehydration, weakness, mental status changes, with a rapid progression to uremia, coma, and death. This complication may occur during the postpartum period when the placenta is no longer shunting calcium to the fetus [3]. 

In untreated patients, the incidence of fetal complications has been reported to be as high as eighty percent. In treated patients, the incidence of neonatal complications is fifty-three percent. Twenty-seven percent to thirty-one percent of cases result in neonatal death [10, 11]. 

Parathyroidectomy is the best therapeutic option for pregnant patients in the first two trimesters. A recent report advocated routine use of the parathyroid aspiration technique for suspected intrathyroidal parathyroid tumors and in ambiguous situations when the identity of the parathyroid gland is in doubt [12]. 

In the patient described, parathyroidectomy with a guide wire technique was performed in the second trimester with no maternal or fetal complications. As compared to the other trimesters, anesthesia risks to infant and pregnant patients are decreased once in the second trimester. Norman and colleagues reported their experience over a period of six years on thirty-two women with sporadic primary hyperparathyroidism with seventy-seven pregnancies. Primary hyperparathyroidism was associated with 3.5 fold increase in miscarriage rates. Thus, parathyroidectomy should be offered in early in the second trimester, resulting in decreased future maternal and fetal complications [13].

While the traditional approach has been bilateral neck exploration with identification of all parathyroid tissue, the recent literature has described numerous benefits of targeted parathyroidectomy, including decreased risk of nerve injury and better cosmesis [14, 15]. These data, together with some studies suggesting shorter operative times and lower cost with equivalent results, make techniques facilitating focused parathyroidectomy extremely attractive. This paper describes a novel, highly successful method of preoperative localization suitable for focused parathyroidectomy [16]. 

The benefits of this technique are apparent. The high degree of accuracy afforded by ultrasonographic examination immediately prior to surgery allows accurate placement of the needle, and then guide wire. Only four similar cases have been described where wire and needle localization was used for surgery, however, all of them utilized CT guidance [17–20]. The overall technique was similar, using image-guidance to precisely place a guide wire into the target lesion closely associated with surrounding structures. The possibility of spread of parathyroid tissue was considered, but there is no current evidence that recurrent hyperparathyroidism has been caused by penetration of an adenoma with a needle. Despite long-term follow-up, needle aspiration has become a standard technique. The ability to follow the wire intraoperatively avoids unnecessary trauma to these other structures from blind dissection, and may alleviate the need for an extensive operation.

This Technique is not necessary for all reoperative parathyroid explorations for recurrent or persistent disease. It was selected specifically for use in these unusual circumstances. It is described as an effective technique for selected high-risk patients.
